# The balance of two opposing factors Mad and Myc regulates cell fate during tissue remodeling

**DOI:** 10.1186/s13578-018-0249-8

**Published:** 2018-09-15

**Authors:** Morihiro Okada, Yun-Bo Shi

**Affiliations:** Section on Molecular Morphogenesis, Eunice Kennedy Shriver National Institute of Child Health and Human Development (NICHD), National Institutes of Health (NIH), 18 Library Dr., Bethesda, MD 20892 USA

**Keywords:** Intestinal development, Adult stem cells, Programmed cell death, Thyroid hormone receptor, Metamorphosis, *Xenopus laevis*, *Xenopus tropicalis*

## Abstract

Cell proliferation and differentiation are two distinct yet coupled processes in development in diverse organisms. Understanding the molecular mechanisms that regulate this process is a central theme in developmental biology. The intestinal epithelium is a highly complex tissue that relies on the coordination of cell proliferation within the crypts and apoptosis mainly at the tip of the villi, preservation of epithelial function through differentiation, and homeostatic cell migration along the crypt-villus axis. Small populations of adult stem cells are responsible for the self-renewal of the epithelium throughout life. Surprisingly, much less is known about the mechanisms governing the remodeling of the intestine from the embryonic to adult form. Furthermore, it remains unknown how thyroid hormone (T3) affects stem cell development during this postembryonic process, which is around birth in mammals when T3 level increase rapidly in the plasma. Tissue remodeling during amphibian metamorphosis is very similar to the maturation of the mammalian organs around birth in mammals and is regulated by T3. In particular, many unique features of *Xenopus* intestinal remodeling during metamorphosis has enabled us and others to elucidate how adult stem cells are formed during postembryonic development in vertebrates. In this review, we will focus on recent findings on the role of Mad1/c-Myc in cell death and proliferation during intestinal metamorphosis and discuss how a Mad1–c-Myc balance controls intestinal epithelial cell fate during this T3-dependent process.

## Background

Cell proliferation and differentiation represent pivotal events for proper organ development and homeostasis. Research on cell proliferation and differentiation has gained considerable recognition because of the promise of cancer therapies. To comprehend the mechanism of cancer, it is necessary to understand how cell proliferation and differentiation are controlled in normal cells and how they are dysregulated in cancer cells. Transcription factors form important links in the coordination of complex biological processes by responding to intra- and extra-cellular signals, recruiting coregulators to target regions, and controlling the rate of transcription of downstream genes. These genes are critically involved in determining cell fate such as cell proliferation, differentiation, and death. One of the best-known transcriptional networks for the control of proliferation, differentiation, and cell growth is Myc/Mad/Max. Among the three members of the Myc oncoprotein family (c-Myc, N-Myc, and L-Myc), c-Myc is the best characterized and encoded by the MYC proto-oncogene, which heterodimerizes with the partner protein Max [1]. Extensive studies have provided strong evidence for the involvement of Myc in tumorigenesis, including carcinoma of lung, breast, and colon., etc. [2–6]. Additionally, a null c-Myc mutation in mice causes embryonic lethality with defects in growth, cardiac and neural development, vasculogenesis, and angiogenesis, suggesting that c-Myc is critical for both development and tumor progression [7, 8]. Contrary to the function of c-Myc in tumor progression, c-Myc has a pivotal function in apoptosis under physiological conditions. It is well known that overexpression of c-Myc can induce apoptosis in normal cells, but not in cancer cells [9–18]. Conversely, upon heterodimerizing with Max, Mad proteins, including Mad1, Mxi1, Mad3 and Mad4, strongly antagonize c-Myc transforming activity, causing cells to exit the cell cycle and enter differentiation and/or growth arrest [19]. Whereas overexpression of c-Myc can induce apoptosis in normal cells, Mad has been most often found to be anti-apoptotic [11, 20–22]. Myc and Mad share the same heterodimeric partner, Max, suggesting that the cell proliferation/differentiation fate is determined by the Myc/Mad/Max network.

Owing to their antagonistic cellular functions, c-Myc and Mad expression is often regulated in opposite manner and spatiotemporally successive or distinct in different tissues. For example, c-Myc expression is high in proliferating stem cells while Mad expression is found to increase when cells undergo differentiation and migrate upward along the crypt–villus axis in the adult mouse intestine [20, 23–25]. Additionally, Mad proteins repress the activation of genes inducible by Myc, prevent cell growth, and block cell death [22, 26]. Given the many reports of the widespread induction of Mad gene expression during terminal differentiation, Mad likely inhibits a number of genes potentially important for cell proliferation. However, the role of the Myc/Mad/Max network during postembryonic development in vertebrates is unclear, because it is hard to study late-stage embryos or neonate in mammals due to the maternal interference and technical difficulties.

## *Xenopus* intestinal metamorphosis as a model to study the control of cell proliferation vs. differentiation during postembryonic vertebrate development

Frog metamorphosis shares many similarities with mammalian postnatal development, including the presence of high levels of plasma thyroid hormone (T3) [27, 28]. During mammalian fetal development, the exposure of fetal tissues to maternal hormones and other factors though the placenta is essential for proper development [29]. Thus, it is difficult to study the effects of hormones such as T3 on fetal development without maternal interference. In contrast, amphibian embryos develop externally in the absence of any maternal influence, and their metamorphosis is absolutely dependent on T3, allowing the process to be manipulated by simply adding T3 to tadpole rearing water or inhibitors to block the synthesis of endogenous T3.

Amphibian metamorphosis involves systematic transformations of various tissues/organs. Of particular interest is the dramatic transformation of the intestine. In *Xenopus*, the tadpole intestine has mainly a monolayer of larval epithelial cells and thin layers of surrounding connective tissue and muscles [30–32]. The larval epithelial cells are fully differentiated into specific cell types yet retain a capacity of, and often undergo, mitotic division [33]. As plasma T3 levels rise, thereby inducing metamorphosis, a de novo formation of proliferating adult epithelial stem cells takes place through the de-differentiation of larval epithelial cells, via a yet-unknown mechanism [30, 34, 35]. Concurrently, the rest of the larval epithelial cells undergo programmed cell death or apoptosis as new adult cells expand to completely replace the dying larval epithelial cells [34]. These findings indicate that morphologically identical larval epithelial cells can choose two mutually exclusive pathways: apoptosis or dedifferentiation into adult stem cells, in response to T3 during metamorphosis [30]. Subsequently, the proliferating adult epithelial cells differentiate into specialized cells to form a more complex adult intestinal epithelium, surrounded by well-developed, thick layers of connective tissue and muscles by the end of metamorphosis [30]. In the multiply folded adult intestinal epithelium, adult stem cells reside in the trough of the fold while differentiated epithelial cells die off mainly at the tip of the fold, resembling the crypt-villus axis in the adult mammalian intestine [30, 36].

## Mechanism of T3 regulation of adult stem cell development

Thyroid hormone can function through both genomic and non-genomic pathways. Its genomic effects are mediated by T3 receptors (TRs) encoded by two genes, TRα and TRβ, that are conserved in all vertebrates. Based on biochemical and molecular properties of TRs and their expression profiles during *Xenopus* metamorphosis, we have previously proposed a dual function model for the role of TRs [37–39]. That is, during premetamorphosis, due to the lack of T3, TRs mainly function as unliganded transcription repressors to repress T3-regulated genes, thus helping to prevent tadpoles from undergoing precocious metamorphosis. During metamorphosis, the presence of high levels of T3 leads to the formation of liganded TR, which in turn activates the very same genes to promote metamorphosis. Indeed, subsequent molecular and transgenic studies have provided strong evidence to support the model and further demonstrated that TR is both necessary and sufficient for mediating the metamorphic effect of T3. More recently, by using gene-editing technology, it has been demonstrated clearly that endogenous TRα functions to prevent precocious metamorphosis by repressing target genes during premetamorphosis and regulates metamorphosis rate when T3 is present during metamorphosis [40–45]. Similarly, knocking out endogenous TRβ also affected metamorphosis, although the effects are much less than knocking out TRα [46, 47].

Like other processes during metamorphosis, T3 signaling is required for intestinal metamorphosis, including the formation of adult intestinal stem cells, and this effect of T3 is mediated by TR. Many studies using *X. laevis* have demonstrated that the proliferating intestinal stem cells are formed de novo via the dedifferentiation of a very small fraction of the larval epithelial cells in a process that requires T3 signaling in both intestinal epithelial and non-epithelial tissues [34, 48]. Furthermore, TR is both necessary and sufficient for mediating the effect of T3 for the formation of such adult stem cells [39, 49]. Thus, to determine the molecular mechanisms underlying adult stem cell formation in the epithelium, it is critical to identify genes that are regulated by T3 in the epithelium as well as in the non-epithelial tissues. A genome-wide microarray analysis of the epithelial and non-epithelial tissues during intestinal remodeling [50] revealed many T3-inducible genes that are likely involved in the formation of the adult stem cells, such as PRMT1 [51], AMDHD1 [52], HAL2 [53, 54], Sox3 [55], and Evi1 [56, 57]. Of particular interest among such candidate stem cell genes is Mad1, which has previously been associated with cell differentiation and anti-apoptotic but not with stem cells [11, 20–22]. Our recent studies reveal an interesting role for Mad1 during intestinal remodeling [58].

## A novel role for Mad1 in T3-induced cell death

**Fig. 1:**
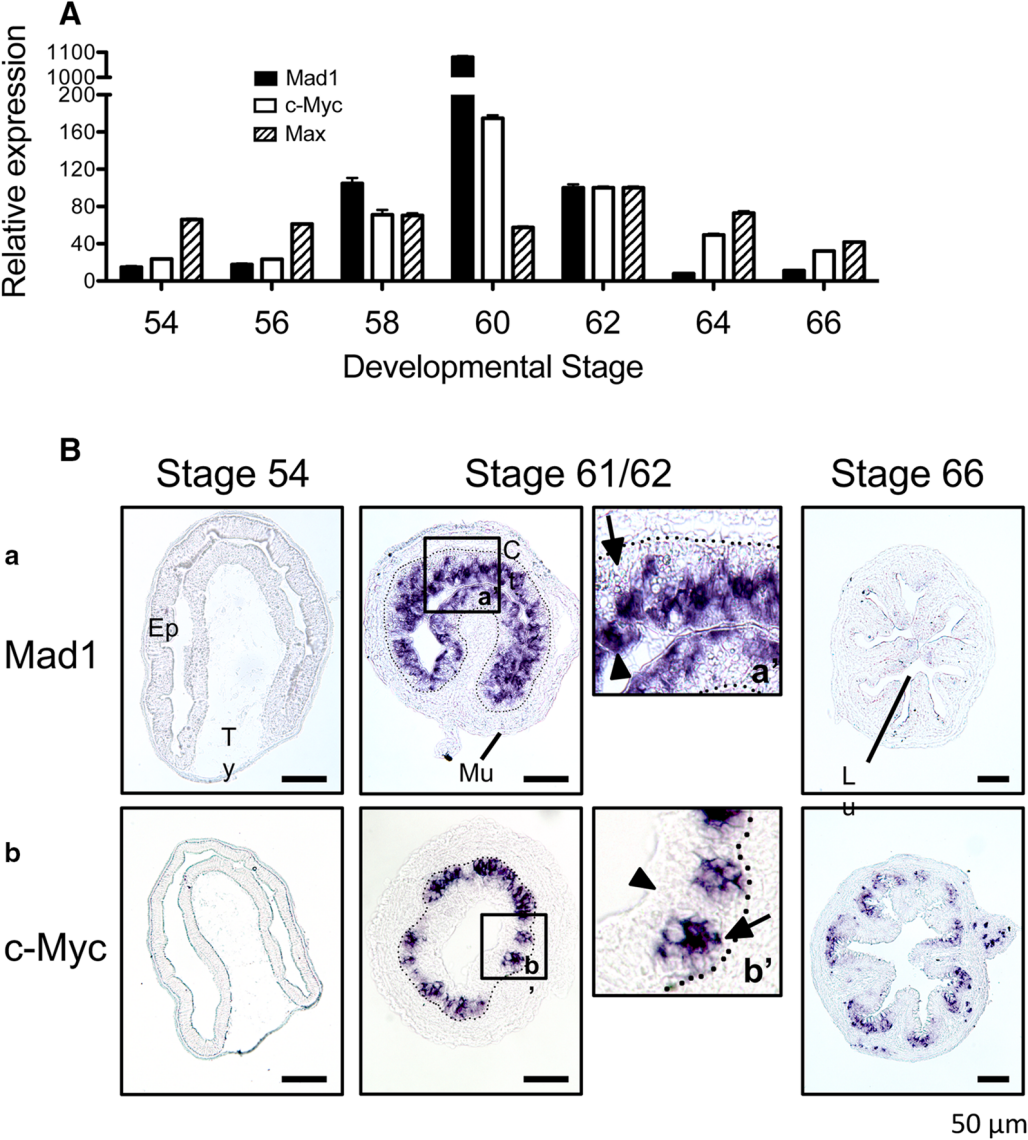
High levels of Mad1 mRNA are present in the degenerating larval epithelial cells facing the lumen while c-Myc is expressed in the proliferating adult cells at the climax of intestinal remodeling. **A** Both Mad1 and c-Myc, but not their dimerization partner Max, are upregulated, during intestinal remodeling. RT-PCR was performed using whole intestine to compare the expression profiles of Mad1, c-Myc, and Max during *X. laevis* intestinal metamorphosis. The mRNA level for Mad1, c-Myc and Max was normalized against that of EF1α RNA. The data are shown in arbitrary unit as the mean ± S.E. (n = 3). See [58] for more details. **B** Mad1 but not c-Myc is expressed in the larval cells undergoing apoptosis at the climax of intestinal remodeling. Cross-sections of *X. laevis* intestine at premetamorphic stage 54, metamorphic climax stages 61/62 and the end of metamorphosis (stage 66) were hybridized with Mad1 (a) and c-Myc (b) antisense probe. The boxed region in (a and b) at stage 61/62 were enlarged and shown in (a′ and b′), respectively. Note that Mad1 expression was limited to dying larval epithelial cells facing the intestinal lumen at the climax of metamorphosis. The expression of c-Myc was also high at the climax of metamorphosis but was in the epithelial layer close to the connective tissue. Arrows point to clusters of cells or islets in the epithelium close to the connective interface and expressing c-Myc, whereas arrowheads point to the epithelial cells facing the lumen, expressing Mad1. The approximate epithelium–mesenchyme boundary was drawn based on morphological differences between epithelial cells and mesenchyme cells in the photographs, under enhanced contrast and/or brightness by using Photoshop, if needed (dotted lines). Scale bar, 50 μm. *CT* connective tissue, *Ep* epithelium, *Mu* muscle, *Lu* lumen, *Ty* typhlosole. See [58] for more details

Developmentally, Mad1 is expressed at a low level in the intestinal epithelium at premetamorphic stages 54–56 but is significantly upregulated during intestinal metamorphosis, and reaches peak levels around stage 60, when plasma T3 level is high and massive apoptosis occurs in larval epithelium [31]. Its expression level drops to a much lower level by the end of metamorphosis (stage 66), when intestinal remodeling is completed and the cell-renewal system is established along the trough-crest axis of adult epithelial fold (Fig. 1A) [58]. Interestingly, the expression of c-Myc, which is an antagonist of Mad1 and known to be a T3-regulated gene during metamorphosis [59], has a similar expression pattern during intestinal metamorphosis (Fig. 1A) [58]. However, the expression of Max, which is the dimerization partner for both Mad1 and c-Myc, changes little during intestinal metamorphosis (Fig. 1A). Such expression profiles suggest that both Mad1 and c-Myc are likely involved in intestinal metamorphosis but raises the question of why the two opposing factors are similarly regulated.

Spatial expression profiles suggest that Mad1 and c-Myc function in distinct cell types. While both have little expression in premetamorphic intestine, high levels of Mad1 mRNA are localized to degenerating cells facing the intestinal lumen at metamorphic climax (stage 61/62) (Fig. 1B) [58]. The c-Myc mRNA is similarly specifically localized to the epithelium of metamorphic climax intestine but importantly in clusters of epithelial cells close to the connective tissue, not facing the lumen (Fig. 1B). Furthermore, these c-Myc positive cells also express PCNA (proliferating cell nuclear antigen), a marker of cell proliferation based on immunohistochemistry, consistent with earlier studies showing that such epithelial clusters are proliferating cells bearing markers of adult intestinal stem cells [33]. In contrast, the Mad1-expressing cells do not have PCNA. Thus, c-Myc is involved in the formation and/or proliferation of adult intestinal stem cells close to the connective tissue, whereas Mad1 participates in the apoptotic degeneration of larval epithelial cells facing the lumen.

Such a potential pro-apoptotic role for Mad1 contrasts with findings from studies in cell cultures or adult tissues, where Mad1 is implicated in cell differentiation and is anti-apoptotic [11, 20–22], suggesting possible tissue- and/or development-specific roles for Mad1. The ability to knockout genes in the diploid anuran species, *X. tropicalis*, which is closely related to *X. laevis* and undergoes essentially identical metamorphic transformation, including intestinal remodeling [32], makes it possible to investigate the physiological role of Mad1 during frog development. Mad1 knockout studies show that Mad1 is not essential for frog development, with animals developing apparently normally at least up to the end of metamorphosis (stage 66). The rate of development and external morphology of the knockout animals are similar to those for the wild type ones based on three criteria: external morphology, body weight, and intestinal length at the end of metamorphosis [58]. Thus, Mad1 does not play an essential role in embryonic development or metamorphosis.

**Fig. 2:**
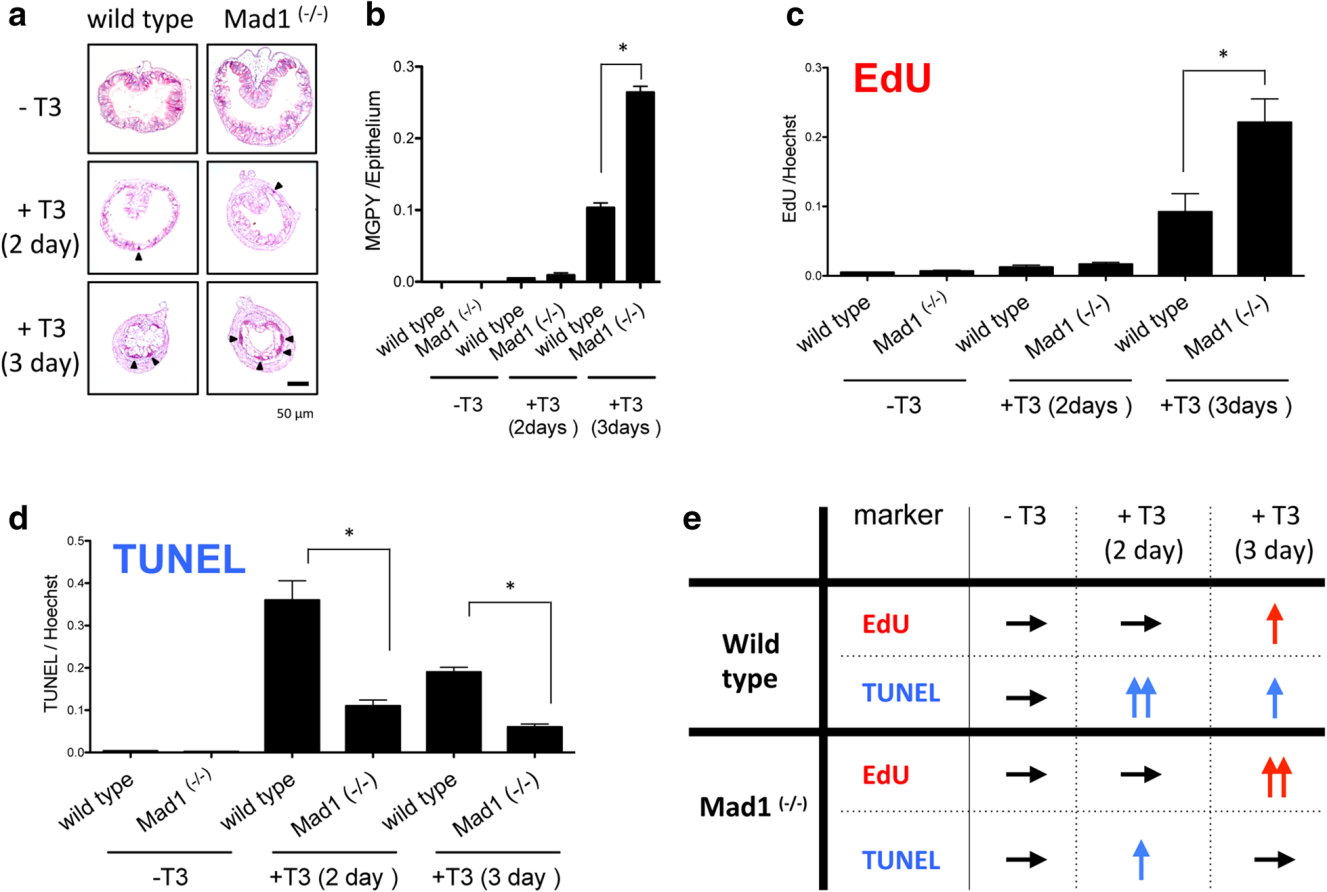
Mad1 regulates developmental cell death and Mad–Myc balance controls the expansion of adult intestinal epithelial cells. **a** Methyl green-pyronin Y (MGPY) staining, which stained the proliferating adult epithelial cells strongly (purple), reveals increased adult stem cell clusters in the knockout animals compared to those in the wild-type tadpoles after 3 days of T3 treatment. Premetamorphic stage 54 tadpoles were treated with 5 nM T3 for 0, 2 or 3 days and were killed 30 min after EdU injection. See [58] for more details. **b** Quantitative analysis of MGPY-positive areas in the epithelium and normalized by the total cellular area in epithelium. The statistical significance of the differences was determined by Student’s *t* test (*P < 0.05). Tadpoles of each genotype (n = 3–5) were used for counting MGPY-positive areas in the epithelium. See [58] for more details. **c** Cell proliferation is significantly increased in Mad1 (−/−) tadpoles treated with T3 for 3 day compared to that in the wild-type ones. Cross-sections of the intestine of the tadpoles above were stained for cell proliferation by EdU staining. EdU positive areas in epithelium of *X. tropicalis* intestine were measured and normalized against the total cellular area in epithelium determined by Hoechst staining. The statistical significance of the differences was determined by Student’s t-test (*P < 0.05). Tadpoles of each genotype (n = 3–5) were used for counting EdU-positive areas in the epithelium. See [58] for more details. **d** More intestinal epithelial cell death in T3-treated wild-type animals than that in Mad1 (−/−) tadpoles. Cross-sections of the intestine of the tadpoles above were stained for apoptosis by TUNEL. Quantitative analysis of apoptosis by counting TUNEL-positive areas in the epithelium and normalized by the total cellular area in epithelium determined by Hoechst staining. The statistical significance of the differences was determined by Student’s t-test (*P < 0.05). Tadpoles of each genotype (n = 3–5) were used for counting TUNEL-positive areas in the epithelium. See [58] for more details. **e** Summary of the changes in cell proliferation (EdU) and apoptosis (TUNEL) in the intestinal epithelium after 5 nM T3 treatment for 0, 2 or 3 days in wild type and Mad1-knockout animals. Note that Mad1 knockout not only enhances the cell proliferation but also reduces cell death in the epithelium during T3-induced metamorphosis

Analysis of the intestine during natural metamorphosis did not reveal obvious difference between wild type and Mad1-knockout animals. This may be due to compensation by other Mad family members and/or asynchrony among tadpoles as they progress through metamorphosis, making it difficult to observe changes caused by the Mad1 knockout. On the other hand, when premetamorphic tadpoles (stage 54) were treated with T3 for 0–3 days to induce metamorphosis, there were more stem cell clusters and more cell proliferation in the epithelium of Mad1 knockout tadpoles than wild-type tadpoles after 3 days of T3 treatment (Fig. 2a–c, e), indicating that Mad1 knockout enhances adult intestinal epithelial stem cell formation and/or proliferation during T3-induced metamorphosis. In addition, an analysis of cell death with TUNEL (terminal deoxyribonucleotidyl transferase-mediated dUTP-biotin nick end labeling) assay revealed that high levels of apoptosis were present in the epithelium of wild-type tadpoles after 2 days of T3 treatment and this cell death was reduced in the Mad1 knockout tadpoles (Fig. 2d, e). Similar reduction in apoptosis in the epithelium was observed after 3 days of T3 treatment, although overall cell death was less compared to that after 2 days of T3 treatment. Thus, these data indicate that Mad1 plays an important role in T3-induced larval epithelial apoptosis.

## A potential mechanism for the myc/mad/max network in regulating T3-dependent intestinal metamorphosis

**Fig. 3:**
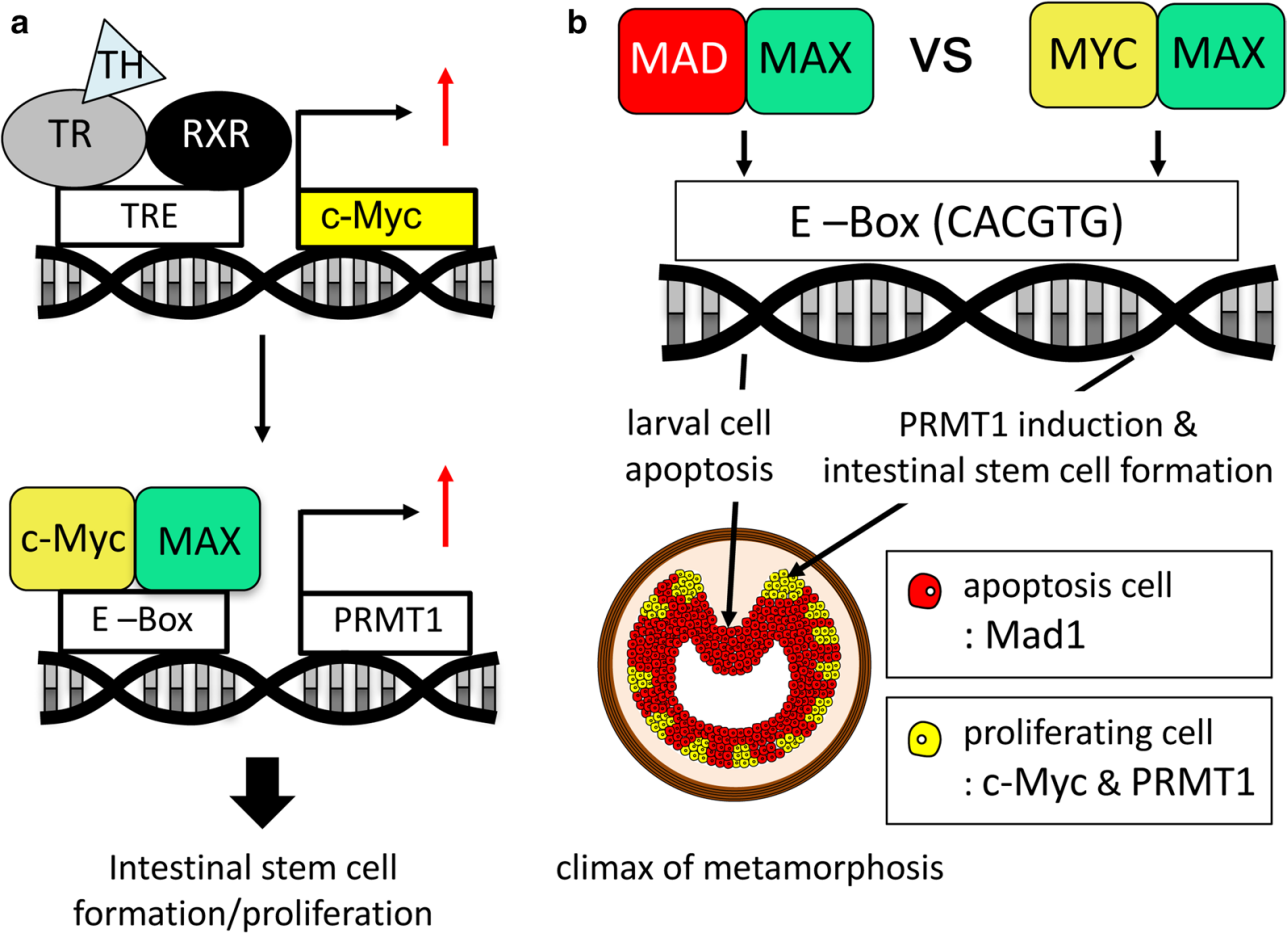
A balance of Mad and Myc controls cell fate determination during intestinal remodeling. **a** For T3-induced genes, TR normally functions as a heterodimer with RXR (9-cis retinoic acid receptor). During metamorphosis, T3 induces the expression of c-Myc directly at the transcription level through TR binding to the TRE in the c-Myc promoter. Then, c-Myc activates the expression of histone methyltransferase PRMT1 through binding to the intronic enhancer in the PRMT1 gene. PRMT1, in turn, participates in the intestinal stem cell formation/proliferation. **b** Both Mad1 and c-Myc can heterodimerize with Max. Thus, Mad1 competes against c-Myc to regulate target gene expression. When high levels of Mad1 are present in a cell, Mad1 causes mitotically active larval epithelial cells to exit the cell cycle to facilitate their degeneration through apoptosis. Whereas, high levels of c-Myc competes again Mad1 to activate PRMT1 expression. PRMT1, in turns, participates in the formation and/or proliferation adult intestinal stem cells. High levels of Mad1-expressing cells, undergoing apoptosis, are indicated by red dots. The c-Myc and PRMT1-expressing cells, which are proliferating adult stem cells, are indicated by yellow dots

As stated above, T3 induces the de novo formation of adult intestinal stem cells. Earlier studies have demonstrated that T3 activates c-Myc expression directly via the binding of TR to the T3 response element (TRE) in the c-Myc promoter region (Fig. 3a) and that c-Myc in turn activates protein arginine methyl- transferase 1 (PRMT1) expression via c-Myc binding to an intronic enhancer in the PRMT1 gene (Fig. 3a) [59]. This may be responsible for the T3 upregulation of PRMT1 expression, which peaks at the climax of intestinal remodeling [60]. More importantly, PRMT1 has been shown to play a pivotal role for either the formation and/or proliferation of the adult epithelial stem cells in the intestine during metamorphosis [51]. The upregulation of the cMyc/Max-PRMT1 pathway is thus at least responsible in part for the T3-induced formation/proliferation of adult intestinal stem cells (Fig. 3a). On the other hand, the upregulation of Mad1 expression by T3 in the larval epithelial cells facilitate their apoptotic degeneration. The simultaneously activation of both Mad1 and cMyc in different subset of epithelial cells thus helps to ensure most of the larval epithelial cells undergo apoptosis. Additionally, a small number of larval epithelial cells, which are induced by T3 to express cMyc, undergo dedifferentiation to become adult stem cells and actively proliferate to form the adult epithelium (Fig. 3b). In Mad1 knockout animals, the lack of Mad1 delays or reduces larval epithelial cell death and thus enables more larval epithelial cells undergo dedifferentiation into adult stem cells. Alternatively, the low levels of Mad1 are expressed in the adult stem cells and their removal in the knockout animals enable the adult stem cells to proliferate faster.

## Conclusion

The mammalian intestine has been investigated extensively as a model for adult organ-specific stem cells due to the high self-renewal rate of the epithelium. This has led to detailed understanding of adult stem cells and their properties, including the identification of a number of signaling pathways important for intestinal development and cell renewal in the adult [61, 62]. On the other hand, much less is known about how the epithelium of the small intestine, which is one of the most architecturally and functionally complex tissues, is formed during development. Amphibian metamorphosis, in particular, intestinal remodeling, provides a unique opportunity to clarify the mechanism of formation of adult organ-specific stem cells during vertebrate development. Increasing evidence suggests that formation of the mammalian adult intestinal stem cells takes place around the neonatal period, which resembles amphibian metamorphosis, and is regulated by T3 [63].

Studies on intestinal remodeling have now revealed a novel role for the Myc/Mad/Max network in T3-induced development of the adult intestine. In particular, unlike earlier studies in adult tissues and cell cultures, Mad1 appears to play a novel role in T3-induced larval epithelial cell death during intestinal metamorphosis. At the same time, cMyc is activated by T3 in a subset of larval epithelial cells, which may facilitate their dedifferentiation into adult intestinal stem cells via transcriptional activation of histone methyltransferase PRMT1. Removal of Mad1 from the larval epithelial cells reduces T3-induced larval apoptosis and increases proliferating stem cells during intestinal metamorphosis, possibly because more larval epithelial cells are now available for dedifferentiation into adult stem cells due to the reduction in larval cell death.

The findings from *Xenopus* metamorphosis model raise a number of interesting questions. First, how does Mad1 affect T3-induced cell death? Mad1 may have two possible functions: direct inducing larval epithelial cell death or indirectly facilitating larval epithelial cell death by causing the cessation of the cell cycle, thus inhibiting proliferation, when T3 is present. Second, it remains to be determined whether the other Mad genes, which have redundant roles as Mad1, participate in frog metamorphosis. Third, what is the mechanism for the selective T3-induced upregulation of Mad1 and c-Myc in distinct epithelial cells? Lastly, despite the rapid progresses in stem cell biology from studies on the adult mammalian intestine, the developmental origin of the adult stem cells remains to be determined. While it has been shown that the adult stem cells in the intestine are formed de novo through the dedifferentiation of some larval epithelial cells during metamorphosis, the mechanism underlying this dedifferentiation is still unclear [34]. It will be interesting to investigate this developmental ‘switch’ induced by T3.
